# Bridging the barrier: insights into blood biomarkers and therapeutic strategies targeting choroid plexus and BBB dysfunction in alzheimer’s disease

**DOI:** 10.1186/s40364-025-00829-4

**Published:** 2025-09-26

**Authors:** Niti Sharma, Danyeong Kim, Himadri Sharma, Moon Il Kim, Hyon Lee, Minju Kim, Nayoung Ryoo, Min Ju Kang, Jung-Min Pyun, Young Ho Park, Jisun Ryu, Hyun Jung Oh, Hyun-Sik Yang, Hang-Rai Kim, Geon Ha Kim, Sangwon Han, YoungSoon Yang, Young Chul Youn, Charlotte Teunissen, Henrik Zetterberg, Philip Scheltens, Seong Soo A. An, Young-Bum Kim, SangYun Kim, the Alzheimer’s Disease All Markers (ADAM) Research Group, Niti Sharma, Danyeong Kim, Himadri Sharma, Moon Il Kim, Hyon Lee, Minju Kim, Nayoung Ryoo, Min Ju Kang, Jung-Min Pyun, Young Ho Park, Jisun Ryu, Hyun Jung Oh, Hang-Rai Kim, Geon Ha Kim, Sangwon Han, YoungSoon Yang, Young Chul Youn, Seong Soo A. An, SangYun Kim

**Affiliations:** 1 Department of Bionano Technology, Gachon Bionano Research Institute, Gachon University, 1342 Seongnam-daero, Sujeong-gu, Seongnam-si, Gyeonggi-do 13120 Republic of Korea; 2 Department of Neurology, Veterans Medical Research Institute, Veterans Health Service Medical Center, 53, Jinhwang-ro 61-gil, Gangdong-gu, Seoul, 05368 Republic of Korea; 3 Department of Neurology, Gachon University Gil Hospital, 21, Namdong-daero 774Beon-gil, Namdong-gu, Incheon, 21565 Republic of Korea; 4 Department of Neurology, Seoul National University College of Medicine & Seoul National University Bundang Hospital, 82, Gumi-ro 173beon-gil, Bundang-gu, Seongnam-si, Gyeonggi-do, 13620 Republic of Korea; 5 Department of Neurology, Eunpyeong St. Mary’s Hospital, The Catholic University of Korea, 1021, Tongil-ro, Eunpyeong-gu, Seoul, Republic of Korea; 6 Department of Research and Development, PeopleBio Inc., 242 Pangyo-ro, 6fl. PDC C-dong, Bundang-gu, Seongnam-si, Gyeonggi-do, 13487 Republic of Korea; 7 Department of Neurology, Brigham and Women’s Hospital/Harvard Medical School, 60 Fenwood Rd 1 Floor, Boston, MA 02115 USA; 8 Department of Neurology, Dongguk University Ilsan Hospital, 27 Dongguk-ro, Ilsandong-gu, Goyang-si, Gyeonggi-do, 10326 Republic of Korea; 9 Department of Neurology, Ewha Woman’s University Mokdong Hospital, Ewha Woman’s University College of Medicine, 1071, Anyangcheon-ro, Yangcheon-gu, Seoul, 07985 Republic of Korea; 10 Department of Neurology, Soonchunhyang University Seoul Hospital, Soonchunhyang University College of Medicine, Seoul, 04401 Republic of Korea; 11 Department of Neurology, Soonchunhyang University Hospital, 31, Suncheonhyang 6-gil, Dongnam-gu, Cheonan-si, Chungcheongnam-do, 31538 Republic of Korea; 12 Department of Neurology, Chung-Ang University College of Medicine, 84 Heukseok-ro, Dongjak-gu, Seoul, 06974 Republic of Korea; 13 Neurochemistry Laboratory, Department of Clinical Chemistry, Amsterdam Neuroscience, Vrije Universiteit Amsterdam, Amsterdam UMC, De Boelelaan 1105, Amsterdam, 1081 HV Netherlands; 14 Department of Psychiatry and Neurochemistry, Institute of Neuroscience & Physiology, the Sahlgrenska Academy at the University of Gothenburg, Mölndal, 43180 Sweden; 15 Clinical Neurochemistry Laboratory, Sahlgrenska University Hospital, Mölndal, 43139 Sweden; 16 Department of Neurodegenerative Disease, UCL Institute of Neurology, Queen Square, London, WC1N 3BG UK; 17 Dementia Research Institute at UCL, Unit 3a, 338 Euston Rd., London, NW1 3BT UK; 18 Kong Center for Neurodegenerative Diseases, Units 1501-1502, 1512-1518, 15/F Building 17W, 17 Science Park W Ave, Science Park, Hong Kong, Hong Kong; 19 Wisconsin Alzheimer’s Disease Research Center, University of Wisconsin School of Medicine and Public Health, University of Wisconsin-Madison, Health Sciences Learning Center, 750 Highland Ave, Madison, WI 53726 USA; 20 Department of Neurology & Alzheimer Center, Amsterdam University Medical Center, Meibergdreef 9, Amsterdam, 1105 AZ Netherlands; 21 Division of Endocrinology, Diabetes, and Metabolism, Beth Israel Deaconess Medical Center and Harvard Medical School, Boston, MA 02215 USA

**Keywords:** Alzheimer’s disease, BBB dysfunction, Choroid Plexus dysfunction, Blood Biomarkers, Early detection, Personalized treatment

## Abstract

Alzheimer’s disease (AD) is the most common cause of dementia and accounts for approximately 60–80% of total dementia patients. Currently, accurate diagnosis for AD relies on cerebrospinal fluid (CSF) sampling or a positron emission tomography (PET) scan, methods that cannot be done in primary care centers where most people go with cognitive complaints. This Limitation calls for the urgent need to develop blood-related diagnostic tests that could facilitate early detection and enable timely treatment. Recent CSF proteomic research categorized AD into five molecular subtypes with discrete Genetic risk profiles. Subtypes 1–3, namely neuronal hyperplasticity, innate immune activation, and RNA dysregulation, were characterized by more classical AD-related changes, like accumulation of amyloid/tau and synaptic and immune dysfunction, respectively. On the contrary, non-traditional AD mechanisms in subtypes 4–5 were choroid plexus (CP) dysfunction and blood–brain barrier (BBB) dysfunction, emphasizing clearance deficits in association with brain barrier dysfunction. The unchanged tau levels later may be explained by an alternate disease mechanism (clearance dysfunction). These subtypes included BBB and CP dysfunction. Biomarker identification based on the mechanism of disease progression would increase the precision of diagnoses, allowing for tailored interventions and aiding in the creation of novel therapies for subtypes that might not react favorably to conventional amyloid/tau-targeting strategies. Finding biomarkers specific to each subtype would aid in patient classification, resulting in more individualized therapy as opposed to a “one-size-fits-all” strategy. The present review emphasized the importance of identifying blood-based biomarkers (BBMs) related to brain barrier dysfunction from CSF studies and personalized treatment strategies to streamline the diagnostic workup, and may be utilized in standard clinical practice for the early detection of AD.

## Introduction

Alzheimer’s disease (AD) is a progressive neurodegenerative disorder (ND) characterized by cognitive decline, memory loss, and behavioral changes. Its pathogenesis is multistep and complex, with deposition of amyloid-β (Aβ) plaques, intracellular neurofibrillary tangles (NFTs) of hyperphosphorylated tau protein, synaptic dysfunction, neuroinflammation, oxidative stress, and eventual neuronal loss [1, 2]. These pathologic alterations primarily involve brain regions critical for learning and memory (hippocampus and cortex), and evolve gradually over the years before the development of clinical symptoms. Currently, ~ 7.2 million Americans aged > 65 are living with AD [3]. From 2000 to 2022, mortalities due to AD almost doubled, increasing by 142% in the United States (U.S.) [3]. Besides its devastating health impact, AD imposed a substantial economic burden on individuals, families, caregivers, and the government. The total cost of caring for people with AD and other dementias in the U.S. is projected to reach $384 billion by year-end and $1 trillion by 2050 [3].

Early diagnosis is critical, yet challenging, since memory decline would be present at the mild cognitive impairment (MCI) stage of AD, with heterogeneity in cognitive decline in each patient. According to a report, 25–30% of symptomatic patients are misdiagnosed with AD in the specialized memory clinics, while this number is 50–70% in the primary/community setting [4]. The rate of misdiagnosis could be reduced by the use of positron emission tomography (PET) imaging or cerebrospinal fluid (CSF) analysis to measure the levels of AD biomarker [5]. Nevertheless, the considerable expenses and invasiveness associated with PET and CSF restricted their effectiveness in identifying pathological markers in clinical settings. Consequently, there is an urgent need to identify reliable, low-cost, and non-invasive blood-based biomarkers (BBMs) for detecting AD. The various blood-based diagnostic kits became commercially available by measuring p-tau181, p-tau217, and Aβ_42_ [5, 6]. Recently, the US FDA approved the first blood-based diagnostic device, Lumipulse G, that aids in AD diagnosis using the p-tau 217/Aβ_42_ plasma ratio [7]. Another commercially available Aβ blood-based early detection kit, ‘AlzOn,’ developed by our group [8], received clearance from the Ministry of Food and Drug Safety, South Korea, CE Marking in the EU, and formal registration with the UK Medicines and Healthcare Products Regulatory Agency.

Lately, AD has been categorized into five subtypes based on CSF proteomic profiling, providing an opportunity to understand the detailed molecular processes of AD subtypes [9]. Subtypes 1–3 (neuronal hyperplasticity, innate immune activation, and RNA dysregulation) were more directly correlated to classical AD hallmarks, such as amyloid/tau accumulation, synaptic dysfunction, and immune response changes. Contrarily, subtypes 4 and 5 [choroid plexus (CP) dysfunction and blood–brain barrier (BBB) dysfunction] were driven more by CSF clearance impairments, correlating with CP and BBB dysfunction rather than primary amyloid/tau pathology [9]. Hence, the non-classical AD subtypes might not be adequately captured by the current disease biomarkers (amyloid, tau, and p-tau). In support of this evolving view, our research group introduced additional promising BBMs beyond classical amyloid and tau, including transactive response DNA-binding protein 43 (TDP-43) [10] and complement component 3 (C3) [11]. TDP-43 has been implicated in tau-independent neurodegeneration, while C3 has a role in neuroinflammation. These findings support the necessity of surpassing the traditional amyloid and tau biomarkers, especially in AD subtypes encompassing CP and BBB dysfunction.

The CP consists of a network of cells that originated from the tela choroidea located in each brain ventricle. Specific regions of the CPP generate and release the majority of CSF in the CNS. The CP in the ventricles serves as a connection between CSF and blood, creating the blood-cerebrospinal barrier (BCSFB). CP is crucial for immune monitoring, CSF production, and BBB maintenance, all of which are closely associated with AD pathology [12]. Recent research indicated the role of CP in regulating the circadian rhythm and neurogenesis [13]. Proteomic analysis of CP revealed the dysregulation of several key pathways in association with protein processing, complement activation, lipids, extracellular matrix, vascular, and mitochondria in early AD [14].

The BBB extends throughout the entire neurovascular unit (NVU) and regulates blood–brain transport. AD progression is closely correlated to the structural integrity of the BBB, which involves a variety of cells and tight junctions. The reduced levels of low-density Lipoprotein receptor-related protein 1 (LRP-1) and increased levels of receptor for advanced glycation end products (RAGE) at the BBB impair Aβ clearance [15, 16]. BBB dysfunction triggers neuroinflammation and oxidative stress, which in turn enhances the activity of β-secretase and γ-secretase, leading to increased Aβ production [17, 18]. The Aβ buildup and ongoing BBB damage may create a vicious cycle, contributing to cognitive decline and the onset of dementia [17]. Recent research indicated that the BBB progressively deteriorates with age in the hippocampus, an essential area for learning and memory. Nevertheless, this faster deterioration is observed in patients with MCI than in the age-matched healthy controls (HC), indicating BBB dysfunction at the early stages of cognitive decline [19].

In addition to BBB and CP, the tanycytes (ependymal cell subtype located in the third ventricle) form a blood-CSF barrier at circumventricular organs (CVOs), brain structures lining the third and fourth ventricles [20]. They regulate body homeostasis based on blood–brain communication [21, 22] and neurogenesis in the hypothalamus [23]. Dysfunction of the BBB and CP seriously impairs the brain environment, affecting tanycytic-mediated neurogenesis. De-regulation of these barriers allows harmful chemicals or inflammatory signals to enter the brain, impeding the action of neural stem cells and tissue repair [23]. Over time, this process became a contributor to AD.

Despite the recent advances in CSF proteomics to categorize AD in five subtypes, including CP and BBB dysfunction, there is still limited research directly connecting these AD subtypes with changes seen in BBMs. In this review, we brought together existing research to explore how certain BBMs might relate to changes in CP and BBB seen in several forms of AD. By integrating evidence from proteomic, imaging, and mechanistic studies, we proposed possible pathways worth further investigation, especially in patients with non-classical AD features.

### Potential blood biomarkers

#### Choroid plexus dysfunction

**Fig. 1:**
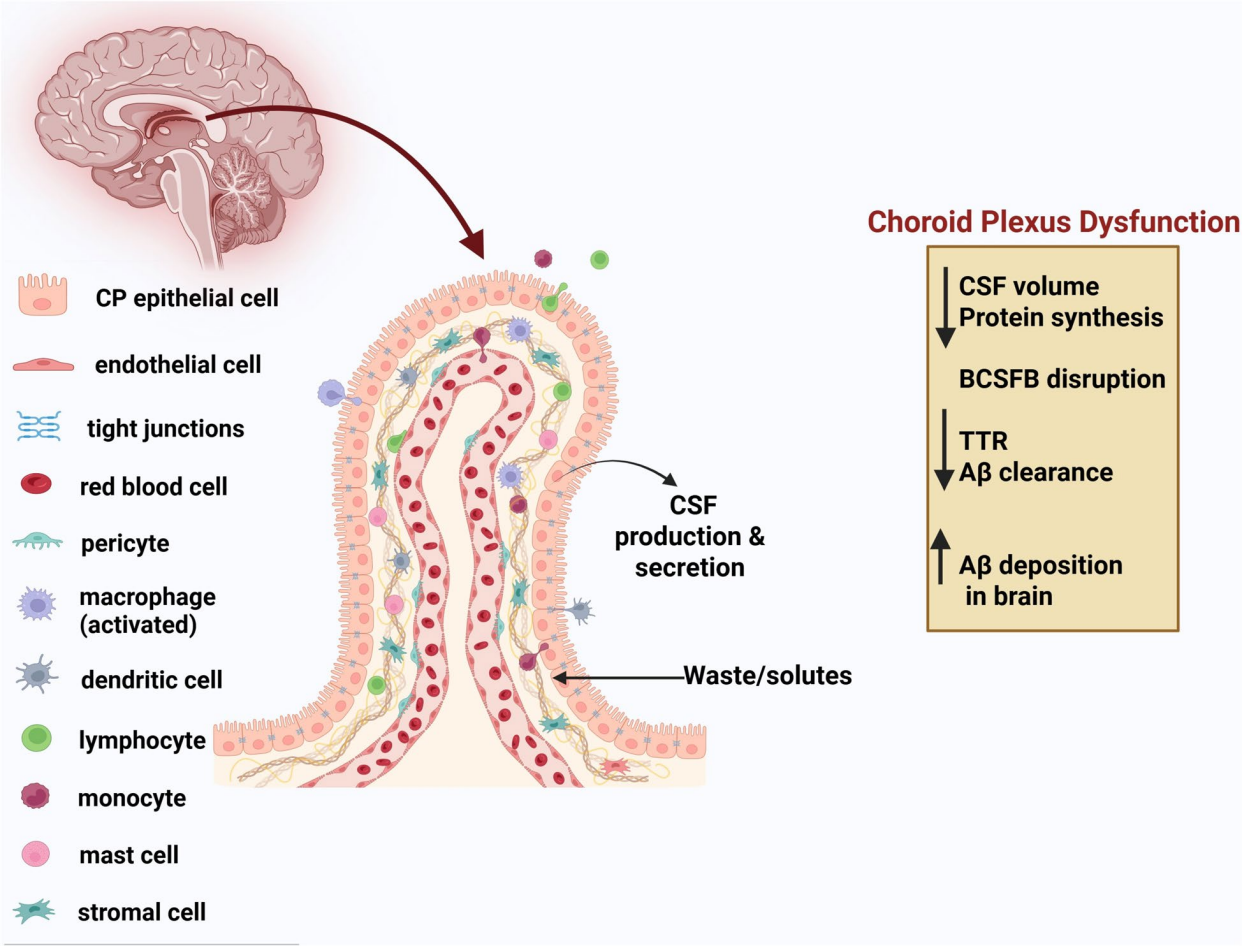
The structure and function of the choroid plexus were impaired in AD. The choroid plexus (CP) consists of a single layer of epithelial cells (CPE) that reside on a basement membrane. The CPE cells were connected by junctional complexes at the luminal membrane, comprising tight junctions. Below the epithelial basement membrane was a network of fenestrated capillaries that were surrounded by connective tissue composed of fibroblasts and immune cells. Cerebrospinal fluid (CSF) was formed and secreted by the ventricles of CP. The epithelial cells constitute the blood-cerebrospinal fluid barrier (BCSFB). Choroid epithelial cells were specialized for transporting hormones and growth factors to the brain through CSF. Dysfunction of CP and BCSFB contributes to age-related cognitive decline and neurodegenerative disease (ND) by decreasing transthyretin (TTR) levels. The compromised TTR expression might lead to Aβ deposition in the brain. Created with BioRender.com

The CP is a highly vascularized structure in the brain’s ventricles responsible for CSF production and maintaining homeostasis. Enlarged CP volume has been reported in patients with AD and inversely correlated with cognitive performance [24–28]. The enlarged volume and increased blood flow to CP could indicate the overcompensation of CSF production in AD [29]. The structure and function of CP are impaired in AD, resulting in secretory, transport, and immune dysfunction and reduced transthyretin (TTR) secretions [30], causing decreased Aβ clearance (Fig. 1**)**.

In a cross-sectional study, participants (N = 108; Age: 22–94 years) from the Baltimore Longitudinal Study of Aging (BLSA) and the Genetic and Epigenetic Signatures of Translational Aging Laboratory Testing (GESTALT) studies were selected to investigate the correlation between CP structure/volume variation and the BBMs (Aβ_42/40_ ratio; p-tau181), neuronal injury (GFAP; NfL), and neuroinflammation. Using advanced magnetic resonance imaging (MRI) techniques, the volume and fine structure (T1 and T2 relaxation times) of CP were measured. The higher levels of p-tau181, NfL, and GFAP were found to be associated with reduced CP integrity [31]. While lower Aβ_42/40_ levels were also correlated with CP changes, significant for one specific MRI measure (T2) [31]. Elevated NfL and GFAP have been associated with neuroinflammation [32, 33], demyelination, and axon degradation [34] in several studies. Previous cohort studies (N = 607; Age: 65.99 ± 8.79 years; ChiCTR2100049131) [24], the AD Neuroimaging Initiative (ADNI; N = 509), and Parkinson’s Progression Markers Initiative (PPMI; N = 302) [35] also identified strong negative associations between CP volume and CSF proteins (Aβ, p-tau, and t-tau) in patients with MCI and AD dementia. These findings suggested that structural deterioration of CP may occur alongside early signs of AD and brain inflammation, even before symptoms appear, emphasizing CP as a promising target for early detection and monitoring of NDs [31].

**Fig. 2:**
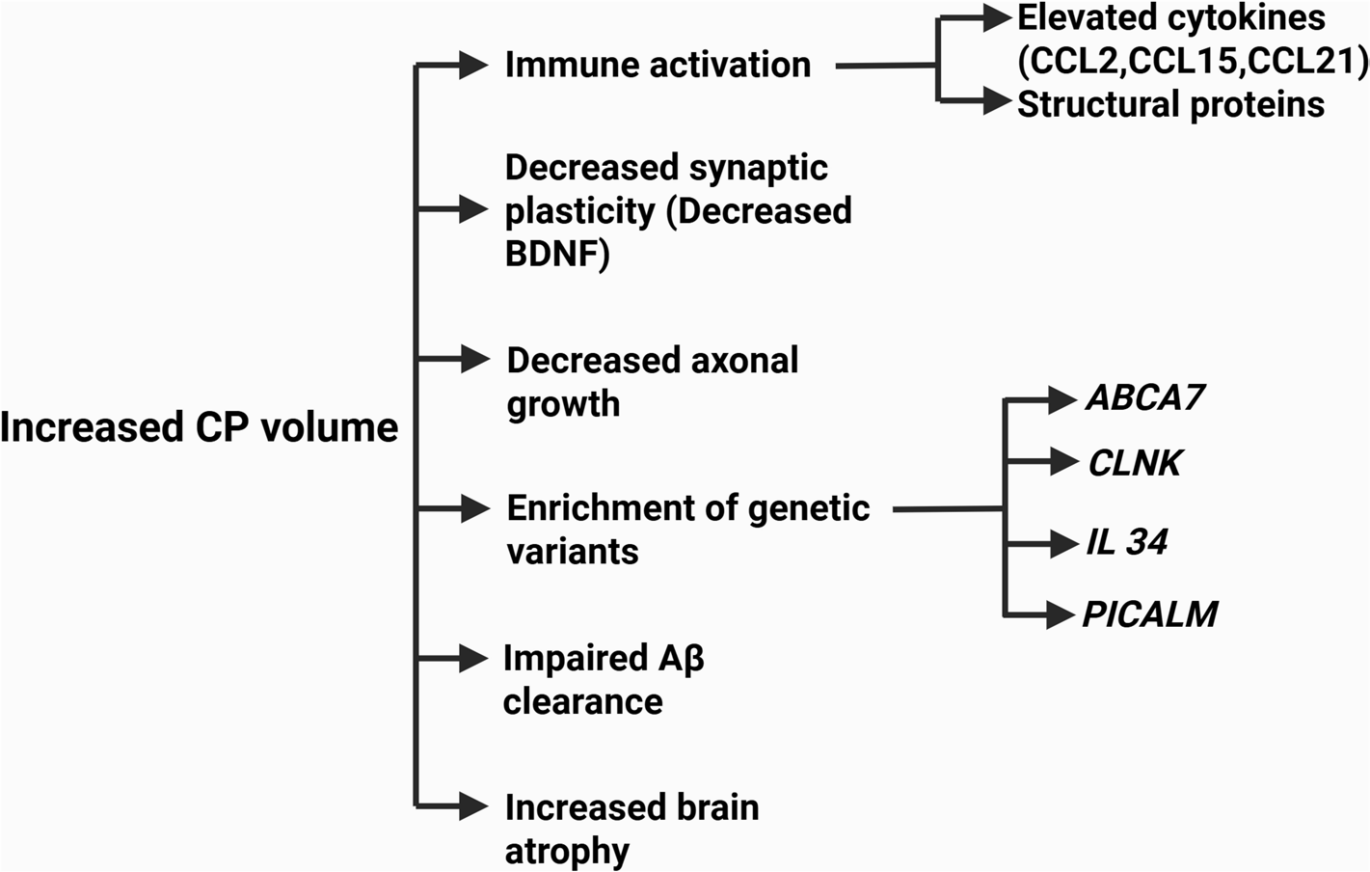
Important indicators and genetic risk factors of CP dysfunction in AD. Created with BioRender.com

The reduced Aβ_40_ and β-secretase (BACE1) levels in this subtype indicated the impaired amyloid clearance mechanisms in the AD pathogenesis. This subtype displayed worse brain atrophy than other subtypes but had normal tau levels [9]. Immune activation was observed due to the elevated levels of cytokines (CCL2, CCL15, and CCL21) and proteins required for the structural support. On the other hand, proteins related to synaptic plasticity (brain-derived neurotrophic factor: BDNF) and axonal outgrowth decreased [9]. When analyzing Genetic risk factors for AD, the enrichments for ATP-binding cassette subfamily A member 7 (*ABCA7*), phosphatidylinositol binding clathrin assembly protein (*PICALM*), interleukin 34 (*IL-34*)*,* and cytokine-dependent hematopoietic cell linker (*CLNK*) were observed [9]. These genes collectively caused AD pathology by impairing Aβ clearance and lipid metabolism and maintaining neuroimmune balance [36, 37] (Fig. 2).

The buildup of abnormal tau in CP is a defining characteristic of AD, leading to its eventual elevation in blood. Nevertheless, the processes involved in removing tau from the brain to the periphery remained uncertain.

The following biomarkers have been reported to be altered in the blood of AD or cognitive impairment patients. Though direct imaging-verified correlations with CP dysfunction are not always present, several of these markers are mechanistically associated with the processes implicated in barrier dysfunction, such as immune dysregulation, disordered transport, and neurovascular injury.

#### Chemokines

Chemokines are heparin-binding proteins (7–15 kDa) involved in chemotaxis. The C–C motif chemokine Ligand 2 (CCL2), also known as monocyte chemoattractant protein-1 (MCP-1), and its receptor, CC-chemokine receptor 2 (CCR2), are implicated in AD [38]. It is involved in clearing Aβ, breaking down myelin, and contributing to neuron loss. Moreover, as CCL2 is found colocalized with amyloid plaques [39], this suggests that CCL2-related inflammation and increased microglial activity may worsen the condition, especially in individuals with defective Aβ processing [40].

In studies conducted on Swedish AD/MCI patients (N = 119; Age: 60–80 years) [40] and (N = 141) [41], elevated plasma and serum CCL2 levels were found compared to those in the HC. In addition, a significant inverse relation was reported between CSF and plasma-CCL2 [41]. In a two-year follow-up study in Taiwan, including AD (N = 310; Mean age: 80.1 ± 7.2 years), MCI (N = 66; Mean age: 75.4 ± 8.2 years), and the HC (N = 120; Mean age: 74.9 ± 7.8 years), a significant correlation between baseline CCL2 and mini-mental state examination (MMSE) score was observed. A higher plasma CCL2 level was associated with greater severity and faster cognitive decline. Additionally, gene polymorphisms (MCP-1-2518A/G and CCR2-190G/A) were associated with higher CCL2 levels in AD [42]. A meta-analysis combining data from several studies showed elevated CCL2, CCL5, and CCL15 in the plasma of AD patients than in the HC [43]. This suggests that the inflammatory processes driving disease progression are primarily confined to the brain rather than occurring systemically.

#### Chitinase-3 Like 1(CHI3L1 or YKL-40)

CHI3L1, a glycoprotein (40 kDa) mainly expressed by astrocytes, is involved in inflammation, innate immunity, and tissue injury/repair/remodeling activities. Upregulation of CHI3L1 was reported in several diseases, including AD, and was correlated with Aβ accumulations, cognitive impairment, and neuroinflammation in the white matter [44–46]. Still, the specific triggers for CHI3L1 production in AD remained ambiguous. Research suggested that Aβ and activated astrocytes may be involved in stimulating CHI3L1 production [47, 48], but further investigations would be needed. A cuprizone-mice (CPZ) study indicated the correlation of CHI3L1 with neuronal degradation in the grey matter, abnormalities in the BBB, and inflammation due to microglial activation in CP [49]. In a clinical study, Korean AD (N = 61, Age: 74.55 ± 1.56 years) and MCI (N = 49, Age: 68 ± 1.00 years) patients were evaluated for plasma CHI3L1 levels in blood using ELISA, and higher concentrations were found in AD than in the HC or MCI patients, and no significant variation in moderate/severe AD [50]. Hence, plasma CHI3L1 could be a potential biomarker for early AD [50]. Although average plasma CHI3L1 levels were elevated in AD, statistically significant differences were also observed between Lewy body dementia (LBD) and HC [51]. In another validation cohort (N = 237; Age: ≥ 60 years) at Washington University Alzheimer’s Disease Research Center (WU-ADRC), the plasma CHI3L1 levels were significantly higher in AD than in the HC and were affected by the age, and not by the sex [48]. Although its levels were higher in individuals with MCI, similar to patterns seen in the CSF, plasma CHI3L1 did not prove useful for predicting disease progression in the study [48]. A meta-analysis of 17 studies (N = 1,811, Age: 60–80 years) with Asian and Caucasian ethnicity concluded that both CSF and plasma CHI3L1 levels are useful tools for early screening and tracking disease progression [52].

#### Glial fibrillary acidic protein (GFAP)

AD patients had persistently higher levels of plasma GFAP, a marker of astrocyte activation, and it became a sensitive BBM for early neuroinflammatory processes. Even in the preclinical phases of AD, a correlation between plasma GFAP levels and amyloid load, cognitive decline, and brain atrophy was observed [53, 54]. Combining plasma GFAP with a basic model (includes age, sex, and APOE ε4 status) in a systematic study (N = 206; Kerr Anglican Retirement Village Initiative in Ageing Health [KARVIAH] cohort) resulted in a higher prediction accuracy. In addition, plasma GFAP and p-tau181 levels increased longitudinally in cognitively unimpaired individuals with amyloid buildup (Aβ +) over a 12-month duration [53]. Plasma GFAP levels revealed significant correlations with CSF GFAP levels, further supporting its validity as a peripheral biomarker of CNS damage. Studies confirmed that plasma GFAP is a stable (~ 7 days at room temperature) and effective biomarker for detecting AD, particularly in more advanced stages (MMSE < 20), and could reliably distinguish AD patients from the HC [55]. These findings strongly suggested its potential diagnostic value and reinforced its role in reflecting underlying astrocytic activation and neurodegeneration.

Although GFAP is mainly associated with parenchymal astrocytic responses, new imaging and histology research suggests a possible connection with CP dysfunction. Recent studies reported a substantial correlation between ventricular expansion and changes in CP volume with plasma GFAP levels in aging [31], where structural changes in CP may reflect a wider pattern of glial activation from the detected GFAP levels. As CP plays a crucial role in brain immune regulation, inflammatory alterations inside or near CP may result in increased levels of GFAP in the blood. These results support the idea that CP dysfunction and astrocyte activation, reflected by higher plasma GFAP levels, may be correlated with the early stages of AD, even though direct research in AD patients is still limited.

#### Interleukin-34 (IL-34)

IL-34, a cytokine secreted mainly by the neurons, is involved in several signaling pathways in cell survival/growth & differentiation/adhesion/migration, cytoskeletal organization, and expression of other cytokines. Besides neurons, IL-34 also exists in ependymal cells lining the ventricular system and in CP and supports microglial function via colony-stimulating factor-1 receptor (CSF1R) signaling [37]. IL-34 is secreted into CSF by both CP epithelial cells (CPEC) and ventricular ependymal cells, supporting its central role in regulating brain-resident myeloid cells, such as microglia [56, 57]. Reduced IL-34 expression has been correlated to behavioral changes and neuroinflammation in AD [37, 58]. IL-34 aids in breaking down toxic oligomeric Aβ, boosting the production of protective molecules, like transforming growth factor beta (TGF-β) and heme oxygenase-1 (HO-1), and reducing oxidative stress [59]. In the AD mouse model, treatment with IL-34 improved the brain’s ability to clear soluble Aβ and led to better learning performance [37]. IL-34 may also strengthen the BBB by increasing tight junction proteins [60]. Recently, in a study, IL-34 levels were evaluated in CSF, plasma, and saliva of patients (N = 230; Karolinska Memory Clinic at the Karolinska University Hospital, Sweden) at different stages of cognitive impairment. The salivary IL-34 levels were found to be elevated in AD than in the HC and were inversely associated with the MMSE scores [61]. However, IL-34 levels were generally under the detection limit in plasma samples. Altogether, these findings suggested that IL-34 could be a valuable new therapy to protect the brain and slow the progression of neurological diseases.

#### Matrix metalloproteinase 9 (MMP-9)

Several studies demonstrated that MMP-9 plays a significant role in CP barrier dysfunction in AD. Aβ_42_ was shown to induce MMP-9 expression in astrocytes [62] and cerebral endothelial cells [63]. Similar effects were observed in the CP epithelial cells, where Aβ_42_ treatment increased MMP-9 protein levels in both cell lysates and culture media [64]. Notably, MMP-9 is co-localized with Aβ deposits within CP, suggesting a direct association between amyloid accumulation and MMP-9 upregulation [64]. This Aβ-induced increase in MMP-9 expression likely contributes to CP barrier disruption through degradation of tight junction proteins, such as zonula occludens-1 (ZO-1), as evidenced by reduced ZO-1 levels and altered epithelial cell permeability [64]. These changes were further associated with enhanced inulin transport across epithelial monolayers, indicating a compromised barrier function. The mechanism may involve Aβ-triggered elevation of nitric oxide (NO) and reactive oxygen species (ROS), which are known to influence MMP-9 activity [65, 66].

The circulating levels of plasma MMP-9 were more elevated in clinical AD (N = 30; Mean age: 71 ± 11 years) than in the HC (N = 32; Mean age: 61 ± 18 years), PD (N = 24; Mean age: 67 ± 10 years), and ALS (N = 35; Mean age: 50 ± 16 years) patients from Massachusetts General Hospital and Weill Medical College of Cornell University [67], which suggested that plasma MMP-9 could be a suitable biomarker to differentiate other NDs from AD.

The study conducted by Tianjin Medical University General Hospital, China (N = 46; Mean age: 68.58 ± 8.04 years) reported higher levels of MMP-9 in neuronally derived extracellular vesicles (NDEV) from AD patient plasma; however, the increase did not correlate with the MMSE scores [68]. On the contrary, increased levels of MMP-9 in serum and CSF correlated with MMSE scores in the study on the Middle East population (N = 56; Mean age: 66.8 ± 7.8 years) [69]. Collectively, these findings implicated MMP-9 as a key mediator of CP epithelial barrier dysfunction in AD [70], contributing to impaired BCSFB integrity and increased neuroinflammation.

#### Neurofilament Light Chain (NfL)

NfL is a recognized biomarker of axonal degeneration in AD and MCI, often increased in CSF and plasma as a representation of compromised neural integrity. Regardless of the etiology, plasma NfL has been associated with the severity of cognitive impairment in MCI or dementia [71, 72]. In the ADNI cohort (N = 243; Average age 75.0 ± 6.6 years), elevated plasma NfL in Aβ + patients was found to be related to glucose hypometabolism, with longitudinal changes limited to cognitively impaired Aβ + individuals [73]. Plasma NfL concentrations were assessed in 543 cognitively unimpaired participants (mean age 69 ± 9 years) from the Arizona APOE Cohort Study. The group included 66 *APOEε4* homozygotes (HM), 165 heterozygotes (HT), and 312 non-carriers (NC). Plasma NfL was also associated with a faster decline in cortical thickness, decreased MMSE score, and *APOE*ε4 allele [74].

Structural neuroimaging identified that structural alterations in CP were directly related to the elevated NfL levels. In a cohort of 108 cognitively unimpaired aging adults aged 22 to 94 years, advanced MRI techniques using quantitative T₁ and T₂ relaxometry identified that individuals with greater plasma NfL levels had larger CP volumes and altered CP tissue features [75]. These findings suggested that the subtle CP changes may be responsible for early neuronal damage.

A new imaging investigation in a cross-sectional analysis (N = 216) from Ruijin NeuroBank of Alzheimer’s Disease and Dementia (RJNB-D) cohort showed that the increased CP free water fraction (FWf; indicator of the barrier and structural degeneration), had a positive correlation with increased NfL levels, suggesting that CP dysfunction might increase axonal damage or inhibit NfL clearance in the neurodegeneration [76]. Thus, CP dysfunction may be one of the mechanisms that alter NfL dynamics (NfL release into CSF and its subsequent transfer to peripheral blood). These findings are indicative of a new view that not only can the CP generate CSF, but it is also most likely to modulate NfL levels through impaired barrier function in AD.

#### Neuronal Thread Protein (NTP)

NTP, especially its AD-related form (AD7c-NTP), is being studied as a potential blood-based and urine-based marker for detecting AD. This protein is found in higher concentrations in the brains of people with AD, particularly in the areas of neurodegeneration. Interestingly, it is not just found in the brain; it can also be detected in urine and CSF [77, 78]. Studies have shown that AD7c-NTP levels are higher in AD, even in MCI, and they tend to go up alongside other markers like tau, which is involved in the formation of tangles in the brain [79–81]. In a study group (52 MCI and 36 HC), Chen et al. reported elevated AD7c-NTP in the serum (as well as CSF and urine) of MCI than in the HC [82].

Though AD7c-NTP is not directly made by CP or BBB, it is likely affected by how these systems function. The dysfunctional CP could lead to a buildup of AD7c-NTP in the brain and CSF, which might then leak into the blood. The BBB, which usually participates as a protective filter between the brain and the blood, also becomes leaky in AD. This may allow proteins like AD7c-NTP to pass more easily into the bloodstream. In this way, higher blood or urine levels of AD7c-NTP may reflect not just brain cell damage but also problems in the brain’s protective and clearance systems. Overall, AD7c-NTP has the potential to be an early and non-invasive marker for AD, indicative of CP and BBB dysfunction. There are no established studies reported on the direct quantification of AD7c-NTP in plasma or serum, likely due to its low concentrations and sensitivity constraints.

#### Transthyretin (TTR)

TTR is a 55 kDa transport protein produced by CPEC and the liver. It is involved in the movement of thyroid hormone precursor (T4) and retinol-binding protein from the blood across CP into CSF [83]. The deterioration of CPECs with age is associated with several NDs, especially AD, as it regulates the removal of Aβ deposits [84].

In the 3xTg-AD mouse, TTR synthesis in CP was significantly reduced, leading to lowered TTR levels in CSF. Since TTR would normally sequester Aβ and prevent its aggregation, this reduction in TTR interferes with Aβ clearance and causes the increased amyloid deposition, notably in the hippocampus and cortex. These mice also displayed worsening of memory impairments, enhanced neuroinflammation, and mitochondrial dysfunction [30]. Similarly, genetic deletion of TTR in mouse models accelerated Aβ plaque deposition and led to earlier cognitive decline, once more supporting its protective role [85]. On the other hand, increased TTR levels, either pharmacologically or genetically, were shown to reduce Aβ deposition, rescue synaptic functions, and normalize memory performances in AD models [86]. These findings suggested that CP dysfunction, through reduced TTR secretion, could take an active role in AD pathogenesis by interfering with Aβ clearance and augmenting neurodegeneration.

In a Taiwanese longitudinal study, a follow-up for 5 years at one-year intervals was conducted on MCI subjects (N = 184, Age: > 60 years). The results indicated a temporal relationship between the plasma TTR level and the conversion from MCI to AD [87]. A study on Korean patients also demonstrated a significant negative correlation between serum TTR in AD (N = 111) and HC (N = 90), while gender/age-related changes were not observed in the study [88]. Ribeiro et al. investigated plasma TTR levels in aMCI (N = 55) and AD (N = 56) patients (Coimbra University Hospital, Portugal) and compared them to the HC (N = 41) at different disease stages and evaluated factors that may affect TTR levels, such as gender, age, and hormones [89]. Both AD and MCI patients had lower levels of TTR than in the HC, suggesting the possibility that TTR could be an early marker for AD. Interestingly, females with MCI or AD had decreased levels of TTR than the males, as well as control females. Levels of TTR in women with AD were correlated with the severity of the disease, while the decreased TTR was not significant in men. The research also did not identify any correlation between levels of TTR and the *ApoE ε4* gene. In addition, TTR in MCI and AD patients had reduced capacity to bind and transport thyroid hormone, and plasma estradiol was reduced in females with MCI and AD. These results suggested that TTR could be used as an early blood biomarker for AD, and disease progression is influenced by sex-related factors [89]. Moreover, regression analyses on a study conducted on European subjects (N = 270), with mild to severe AD, in AddNeuroMed studies and the Alzheimer’s Research Trust cohort and Kings College London (KCL-ART) cohort predicted cognitive decline over the subsequent 6 months [90], suggesting the potential of plasma TTR to serve as a biomarker of the progression and severity of AD.

#### Transmembrane protein 106B (TMEM106B)

It is a glycosylated protein that controls the size and number of lysosomes, motility, trafficking, acidification, and maintains CP integrity. It also aids the neurons to maintain cellular homeostasis, and any impairment in lysosomal transport could result in neurodegeneration. A multicenter Mendelian randomization (MR) study analyzed proteomic data from plasma and CSF across thousands of individuals (European Alzheimer’s and Dementia Biobank consortium, GWAS, and FinnGen cohorts) [91]. It found that higher plasma TMEM106B levels were significantly associated with increased risk of AD [91]. In a proteomic analysis comparing CP from AD, epilepsy, and control subjects, TMEM106B emerged as one of the most Markedly upregulated proteins in AD. It showed an approximately 3.5-fold increase compared to non-AD controls, especially in the cells lining CP [92]. This increase may be a sign of cell stress or problems with lysosomes, which are responsible for breaking down waste in cells. Since TMEM106B aids in controlling lysosome size and function, over-concentrations might lead to CP dysfunction, which could affect how the brain clears waste proteins, makes CSF, and maintains its protective barriers. TMEM106B has the potential to be recognized as a biomarker for AD.

The formation of filamentous, ring-like structures in CP, called Biondi bodies, has also been associated with many NDs. A recent study reported a direct correlation between Biondi bodies and TMEM106B [93]. These aggregates were also stained by several tau-PET ligands, producing off-target signals in PET scans. Hence, the results indicated that TMEM106B amyloids in CP might have influenced the interpretation of the tau PET scan by creating nonspecific signals instead of tau-related pathology [94].

The summary of possible blood biomarkers for CP dysfunction has been listed in Table 1.

**Table 1:** Potential Blood Biomarkers for Choroid Plexus Dysregulation

Biomarker	Gene	Expression & Localization	Function	Molecular Class	Trend in AD	Assay	Evidence Type	Cohort/Study	Participants	Age (yrs)	Ref
CCL2	*CCL2*	Plasma, CSF; localized with amyloid plaques	Monocyte chemotaxis, neuroinflammation	Cytokine	↑ Plasma/Serum	ELISA	Indirect (associated with cognitive decline, Aβ processing)	Swedish, Taiwanese cohorts	310 (AD), 66 (MCI), 120 (HC)	60–80	[39–43]
CHI3L1 (YKL-40)	*CHI3L1*	Astrocytes, white matter	Neuroinflammation, tissue remodeling	Glycoprotein	↑ Plasma	ELISA	Indirect (linked to CP inflammation, Aβ pathology)	WU-ADRC, Korean, Meta-analysis	61 (AD), 49 (MCI), 237; 1811 (meta; AD, MCI, HC)	60–80	[47–52]
GFAP	*GFAP*	Astrocytic cytoplasm	Astrocyte activation	Intermediate filament	↑ Plasma	SIMOA, Olink™	Indirect (linked to CP volume changes via MRI)	BLSA, GESTALT, KARVIAH	108 (MRI), 206 (HC and Aging)	22–94	[31, 53–55]
IL-34	*IL34*	Neurons, CP epithelial cells	Microglial survival, immune modulation	Cytokine	↑ Saliva (not detectable in plasma)	ELISA	Indirect (secreted by CP cells, immune support)	Karolinska Memory Clinic	230 (AD, MCI, HC)	≥ 60	[37, 56–61]
MMP-9	*MMP9*	Astrocytes, endothelial & CP epithelial cells	Extracellular matrix degradation	Metalloproteinase	↑Plasma, NDEVs	ELISA	Indirect (CP tight junction disruption via Aβ induction)	Massachusetts GH, Weill Cornell	30 (AD), 32 (HC), 24 (PD), 35 (ALS)	50–71	[62–70]
AD7c-NTP	*—*	Neurons (CSF, urine, serum)	Neuronal loss & synaptic disconnection	Cytoskeletal protein	↑ Serum	ELISA	Indirect (barrier leakage hypothesis)	Chen et al	52 (MCI), 36 (HC)	Not Specified	[77–82]
NfL	*NEFL*	Axons (CNS, PNS)	Axonal degeneration	Cytoskeletal protein	↑ Plasma	SIMOA	Direct (associated with CP MRI alterations)	RJNB-D, Arizona	543 (HC), 216 (APOE e4 carriers)	22–94	[71–76]
TTR	*TTR*	CPECs, liver	Aβ binding and clearance	Transport protein	↓ Serum/Plasma	ELISA, RID	Indirect (secreted by CP, declines with age/AD)	Taiwan, Korean, European	184(MCI), 111(AD), 270 (HC)	> 60	[30, 84–90]
TMEM106B	*TMEM106B*	Lysosomes, CP epithelial cells	Lysosomal trafficking and stress response	Glycoprotein	↑ Plasma	Proteomics	Indirect (CP upregulation, Biondi body link)	EADB, GWAS, FinnGen	AD	—	[91–94]

*AD* Alzheimer’s disease, *BLSA* Baltimore Longitudinal Study of Aging, *CP* Choroid plexus, *EADB* European Alzheimer & Dementia Biobank, *ELISA* Enzyme-linked immunosorbent assay, *GESTALT* Genetic and Epigenetic Signatures of Translational Aging Laboratory Testing, *GWA*: Genome-wide association studies, *HC* Healthy control, *MCI* Mild cognitive impairment, *PD* parkinson’s disease, *IMR* Immunomagnetic reduction, *KARVIAH* Kerr Anglican Retirement Village Initiative in Aging Health, *NDEs* Neuronally derived extracellular vesicle, *RID* Radial immunodiffusion, *RJNB*-DRuijin NeuroBank of Alzheimer’s Disease and Dementia, *SIMOA* Single molecular arrays, *WU-ADRC* Washington University (WU) Alzheimer’s Disease Research Center. ^1^Information obtained from The Human Protein Atlas

### Blood–brain barrier dysfunction

**Fig. 3:**
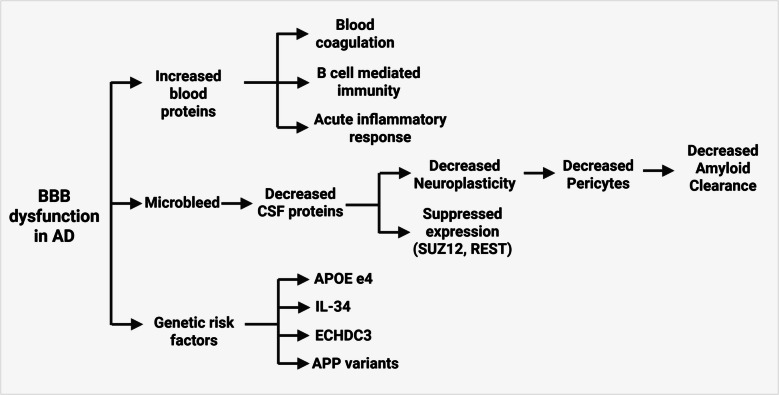
Important indicators and genetic risk factors of BBB dysfunction in AD. Created with BioRender.com

The BBB plays an important role in the maintenance of brain homeostasis by controlling the passage of molecules from the blood into the brain. It also controls the influx of peripheral factors (e.g., cells and molecules) into the CNS and, in part, Maintains a highly defined microenvironment for appropriate neuronal Functions. BBB dysfunction in NDs is characterized by increased BBB permeability, microbleeds, impaired glucose transport, impaired P-glycoprotein 1 function, perivascular deposits of blood-derived products, infiltration of cells, and degeneration of pericytes and endothelial cells [95]. AD subtype with BBB dysfunction had normal levels of CSF tau, decreased BACE1 activation, widespread atrophy, and increased axonal damage [9]. Elevated levels of blood proteins (albumin, fibrinogens, prothrombin, etc.), immunoglobulins (IgG1), inflammation, B-cell activation, blood coagulation-related processes, lipid processing, and lipoprotein clearance were observed in this subtype [9, 96]. Dysregulated plasticity proteins were associated with aberrant gene repression of SUZ12 polycomb repressive complex 2 subunit (*SUZ12*) and RE1-silencing transcription factor (REST) signaling, suggesting hypoplasticity in this AD subtype [9]. Genetically, this subtype showed the highest proportion of individuals carrying the *APOE ε4* allele. In addition, this group was enriched for variants in *IL-34*, *APP*, and enoyl-CoA hydratase domain containing 3 (*ECHDC3*). Notably, IL-34 was also associated with CP dysfunction AD-subtype, suggesting it may contribute to AD through mechanisms involving brain barrier dysfunction and immune regulation. Mutations in the *APP* gene lead to increased Aβ production, which promotes amyloid deposition in cerebral vasculature and triggers downstream inflammatory signals and vascular damage [97]. ECHDC3 has been associated with pathways connected to lipid metabolism and immune signaling in microglia, suggesting a role in regulating microglial clearance and inflammatory activity in the AD brain [9]. Together, these genetic factors present the connection between immune dysregulation, lipid imbalance, and vascular pathology in AD (Fig. 3).

**Fig. 4:**
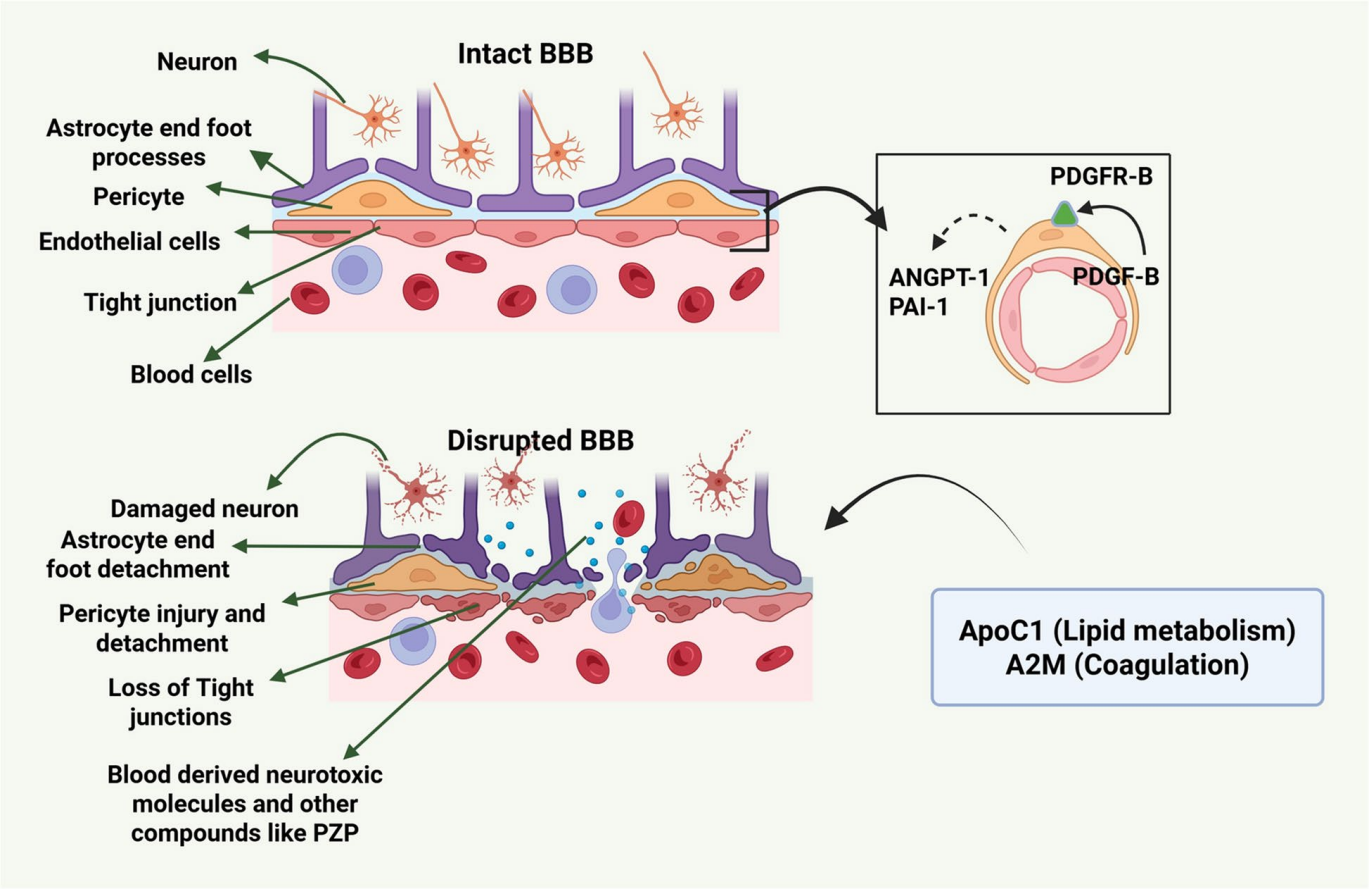
Blood–brain barrier integrity under physiological and pathological (AD) conditions. The structural and functional integrity of BBB was vital to preserving the brain microenvironment homeostasis. BBB was mainly composed of endothelial cells, pericytes, and astrocyte end feet. The tight junctions limit paracellular permeability. To promote pericyte vascularization under physiological conditions, endothelial cells release platelet-derived growth factor-beta subunit (PDGFβ) that binds to PDGFRβ at the surface. Angiopoietin-1 (ANGPT-1) and plasminogen activator inhibitor type 1 (PAI-1) secretion from pericytes endorses the growth of vascular endothelial cells and contributes to the maintenance of BBB. Pathological conditions associated with BBB disruption result in loss of pericytes and tight junctions, compromising BBB integrity. Abnormal expressions of apolipoprotein C1 (ApoC1) and α−2-macroglobulin (A2M) were observed in AD. Elevated levels of A2M were observed in a leaky BBB. The overabundance of A2M could slow down vascular endothelial cells'healing, resulting in blood components penetrating the brain. This increase in blood components may result in inflammation and neuronal death. Created with BioRender.com

The BBB is necessary to protect and regulate cerebral hemostasis. A complex coordinated connection between the endothelial cells covering the blood vessels and the pericytes surrounding them would be required to maintain the integrity of the BBB. The loss of pericyte coverage is a characteristic feature of aging and was a key risk factor for dementia [98]. Morphological changes and fewer pericytes were observed at the site of BBB leakage. Pericyte degradation also enhanced the fibrinogen depositions that damage myelin, oligodendrocytes, and neuronal axons [99]. The endothelial cells secrete platelet-derived growth factor receptor beta (PDGFR-β), which binds to the receptor (PDGF β-R) on pericytes and aids their recruitment during angiogenesis [100] **(**Fig. 4**)**. The subsequent biomarkers have been reported to be modified in the blood of patients with AD or cognitive impairment. While correlations verified by direct imaging with BBB dysfunction are not available for all, many of these markers are connected indirectly to processes involved in barrier dysfunction.

### Apolipoprotein C1 (APOC1)

APOC1 is a protein component of lipoproteins with a role in lipid metabolism, inflammation, and immune response. *ApoC1* is overexpressed in the brain tissue and CSF in AD and is co-localized with Aβ in senile plaques [101]. When present alongside *APOE ε4*, the *ApoC1* insertion allele (rs11568822) acts as an additional risk factor for AD. Individuals carrying both variants show a ~ 66.5% higher risk of developing AD compared to those without this genetic combination [102]. ApoC1 is secreted by activated microglia and astrocytes, particularly around regions of neuroinflammation and BBB disruption. Studies revealed that higher levels of ApoC1 may amplify inflammatory signaling and hamper endothelial cell regenerative capacity, thereby reducing BBB integrity [103]. This may allow the entry of neurotoxic components into the brain, creating a cycle of inflammation, neuronal injury, and cognitive dysfunction. Further, ApoC1 is involved in the modulation of brain cholesterol metabolism [104], which is vital for endothelial cell viability and tight junction integrity. Altogether, these data indicate that ApoC1 has a role in lipid metabolism, and its connection to ApoE suggests a potential indirect influence on BBB health in the context of AD.

A LC–MS-based plasma protein panel (N = 192, Mean age: 75.8 ± 7.3 years) in the ADNI cohort observed a significant decrease in plasma ApoC1 levels in MCI and AD male patients without a significant change in the female group [105]. This study focused on ApoC1’s dual role in lipid metabolism and neuroinflammation, two central inducers of BBB dysfunction in AD. However, the hormonal, genetic, and immunometabolic differences between sexes likely influence its detectability in females, which needs further investigation.

### Alpha-2-macroglobulin (A2M)

The acute-phase protein A2M is a major component of the innate immune system. It is a broad-spectrum protease inhibitor and can inhibit the coagulation process by binding and degrading thrombin [106]. While moderate A2M activity regulates clot formation during BBB repair, excessive levels may impair endothelial healing. This delayed repair can result in BBB leakage, allowing blood components to enter the brain, which in turn triggers inflammation and neuronal damage. Elevated plasma A2M has also been reported alongside markers of BBB disruption, supporting its correlation to vascular dysfunction in AD [105]. Altered levels of A2M in blood were reported in the ADNI (N = 566) and the BLSA (N = 47) cohorts [107], where the elevated A2M levels in plasma were observed in individuals with MCI and AD compared to HC. Moreover, longitudinal studies indicated that higher baseline A2M levels were associated with an increased risk of developing MCI and AD over time [107]. In another ADNI cohort (N = 192; Mean age: 75.8 ± 7.3 years), the levels of plasma A2M were found to be elevated in male AD patients, but not in MCI, compared to the control [105].

A2M correlated with CSF biomarkers (t-tau and p-tau) of neuronal damage and risk of AD progression in cognitively normal individuals within the BIOCARD cohort (N = 303) [107]. Moreover, higher baseline serum A2M levels were independently associated with approximately a threefold risk for developing AD in men [107]. Interestingly, A2M gene and protein expression in brain tissue correlated highly with the levels of calcineurin and RCAN1 (regulator of calcineurin 1), a recognized calcineurin inhibitor and essential phosphatase in dephosphorylation of tau [107]. Together, these findings suggest that A2M is not only associated with preclinical AD and early neuronal injury, but may also be functionally associated with tau phosphorylation dynamics via the RCAN1–calcineurin signaling pathway.

### Angiopoietin-1 (Ang-1)

Ang-1 is an oligomeric glycoprotein with a vascular protective function by inhibiting vascular inflammation, plasma leakage, and preventing endothelial death [108]. Several studies have reported elevated serum Ang‑1 in AD. In one such study involving 42 AD patients, 20 MCI patients, and 40 controls, Ang‑1 was significantly higher in AD and correlated inversely with cognitive function on the MMSE scores [109]. Its upregulation in AD may reflect an endogenous compensatory response to repair BBB damage and maintain vascular integrity. The correlation between elevated serum Ang‑1 and cognitive decline further supports its function as a vascular biomarker of AD.

In animal studies, upregulation of Ang-1 accelerated Aβ_42_ secretion through activation of the forkhead box protein 2/presenilin enhancer protein 2/amyloid precursor protein (FOXA2/PEN2/APP) pathway [110]. As Aβ_42_ deposition was found to cause endothelial cell damage and compromised BBB integrity, elevated Ang-1 levels may reflect ongoing vascular stress and BBB dysfunction. Therefore, increased circulating Ang-1 may not only serve as a marker of neurovascular decline but also as a marker of continuing amyloidogenic activity with BBB disruption in AD.

### Plasminogen activator inhibitor type 1 (PAI-1)

PAI-1, a primary inhibitor of tissue plasminogen activator (tPA), plays a significant role in the regulation of the fibrinolytic and BBB integrity that relies on the plasminogen/plasmin system. In AD, several studies reported the elevated plasma/serum PAI-1 levels, with progressively increasing concentrations from MCI to AD, and these elevated levels were inversely related to cognitive function. In the Ansan Geriatric (AGE) cohort study (N = 226), elevated plasma PAI-1 was associated with increasing disease severity and was significantly higher in MCI as well as AD than in the HC [111]. In the Czech Brain Aging Study cohort (N = 90), elevated serum PAI-1 and an elevated PAI 1/tPA ratio in AD and aMCI patients were observed, which also correlated inversely with the cognitive scores [112]. Increased PAI-1 suppresses tPA, which reduces plasmin formation and clearance of Aβ, a critical pathological feature in AD [113]. In addition to its implication in amyloid metabolism, PAI-1 has been implicated in BBB dysfunction [114]. When PAI-1 levels are too high, it reduces the breakdown of blood clots (fibrinolysis), which can prevent tight junction repair and lead to leaky blood vessels. A consideration of the plasminogen activator system in AD emphasized that elevated PAI-1 is accountable for BBB permeability changes in neurodegenerative pathology. In the same cohort, higher PAI-1/BDNF in AD and its negative correlation with the MMSE score were noticed [115]. An increased PAI‑1/BDNF ratio in AD reflects impaired fibrinolysis and reduced neurotrophic support, essential to BBB integrity. High PAI‑1 inhibits plasmin-mediated tight junction repair, while reduced BDNF diminishes endothelial resilience. Together, this imbalance was likely to enhance susceptibility to these pathologies and correlated PAI‑1/BDNF ratio with BBB dysfunction and cognitive decline in AD patients.

### Platelet-derived growth factor receptor-β (PDGFRβ)

PDGFRβ is expressed by pericytes and arterial vascular smooth muscle cells (VSMCs). In AD, the soluble PDGFRβ (sPDGFRβ) has been reported to be increased in CSF and serum, reflecting damage to pericytes and disruption of the BBB in a murine model [116]. However, information on human studies evaluating serum/plasma PDGFRβ in AD is lacking. In two separate studies (N = 78; Age 23–84 years) [117], and Chongqing Ageing & Dementia Study (CADS) cohort (N = 87; Age: 40 years and over) [118], CSF sPDGFRβ was significantly higher in AD and correlated with CSF albumin (a marker of BBB integrity), total tau, and phosphorylated tau (markers of neuronal injury). These findings suggest that PDGFRβ is associated with pericyte dysfunction and vascular injury, and early cognitive decline in AD.

### PZP alpha-2-macroglobulin-like (Pregnancy zone protein; PZP)

Preclinical AD serum samples from the Rotterdam Scan Study (RSS) (N = 1077; Age: 60–90 years) found that individuals who developed AD had significantly higher serum levels of PZP than the cognitively normal controls [119]. Higher PZP protein and mRNA expressions were also found in postmortem AD brain (N = 20; Netherlands Brain Bank), including microglia and senile plaques, pointing to CNS origin and transport into blood [120]. As a large protease inhibitor with structural similarity to α2 macroglobulin, PZP may play a chaperone-like role in the brain, stabilizing misfolded proteins, such as Aβ [121]. While direct evidence for involvement of PZP in BBB permeability is limited, its accumulation in the brain and passage into the blood, especially early in disease, suggests that BBB dysfunction may permit CNS-derived PZP leakage. In this context, elevated plasma/serum PZP levels are likely to reflect neuroinflammation, amyloid stress, and compromised BBB. The possible blood biomarkers for BBB dysfunction were summarized in Table 2.

**Table 2:** Potential Blood Biomarkers for BBB Dysfunction

Protein	Gene	Protein Expression & Localization	Function	Molecular Class	Trend in AD	Assay	Evidence type	Cohort/Study	Participants	Age (yrs)		Ref	
Apolipoprotein C1	*APOC1*	Secreted to the blood	Lipid transport, LDL inhibition	Lipid transporter	↓ Plasma	LC/MS	Indirect (linked to vascular dysfunction)	ADNI	192 (MCI, AD)	75.8 ± 7.3		[101–105]	
Alpha-2-macroglobulin	*A2M*	Plasma	Protease inhibitor	Serine protease inhibitor	↑ Plasma	LC/MS	Indirect (elevated in AD, may reflect vascular compensation)	ADNI, BLSA, BIOCARD	566 (AD, MCI), 47 (BLSA), 303 (BIOCARD)	60–80 +		[105–107]	
Angiopoietin-1	*ANGPT1*	Plasma	Vascular maintenance, angiogenesis	Developmental protein	↑ Serum	ELISA	Indirect (co-localized with Aβ, associated with BBB disruption)	NINCDS-ADRDA	42 (AD), 20 (MCI), 40 (HC)	> 60		[109, 110]	
Plasminogen activator inhibitor-1 (PAI-1)	*SERPINE1*	Plasma	Inhibits fibrinolysis	Serine protease inhibitor	↑ Serum (PAI-1/BDNF ratio)	ELISA	Indirect (reduces Aβ clearance, affects tight junctions)	AGE Czech Brain Aging Study	226 (AGE), 90 (Czech)	≥ 60		[111–115]	
Platelet-Derived Growth Factor Receptor Beta (PDGFRβ)	*PDGFRB*	Endothelial cells, pericytes	Pericyte recruitment, vascular integrity	Kinase, receptor	↑ Serum	Multiplex fluorescent assay	Direct (correlates with CSF tau/albumin, BBB breakdown)	ADS, CSF studies	87 (CADS), 78 (CSF studies)	23–84		[116–118]	
Pregnancy Zone Protein **(**PZP)	*PZP*	Plasma	Protease inhibition, Aβ binding	Serine protease inhibitor	↑ Serum	Nano LC–MS	Indirect (BBB leakage marker in preclinical AD)	RSS, Netherlands Brain Bank	1077 (RSS), 20 (brain tissue)	60–90		[119–121]	

*ADNI* Alzheimer’s Disease Neuroimaging Initiative, *ADS* Ageing & Dementia Study, *AGE* the Ansan Geriatric cohort, *BBB* Blood–brain barrier, *BDNF* Brain-derived neurotrophic factor, *BIOCARD* Biomarkers of cognitive decline among normal individuals, *BLAS* Birjand Longitudinal Aging Study, *CADS* Chongqing Ageing & Dementia Study, *ELISA* Enzyme-linked immunosorbent assay, *HC* Healthy control, *LC/MS* Liquid chromatography-mass spectrometry, *MS* Mass spectroscopy, *MCI* Mild cognitive impairment, *Nano LC* Nano-liquid chromatography-mass spectrometry, *NINCDS-ADRDA* National Institute of Neurologic and Communicative Disorders and Stroke and the Alzheimer′s Disease and Related Disorders Association, *RSS* Rotterdam Scan Study. ^1^Information obtained from The Human Protein Atlas

### Role of tanycytes in CP and BBB dysfunction

**Fig. 5:**
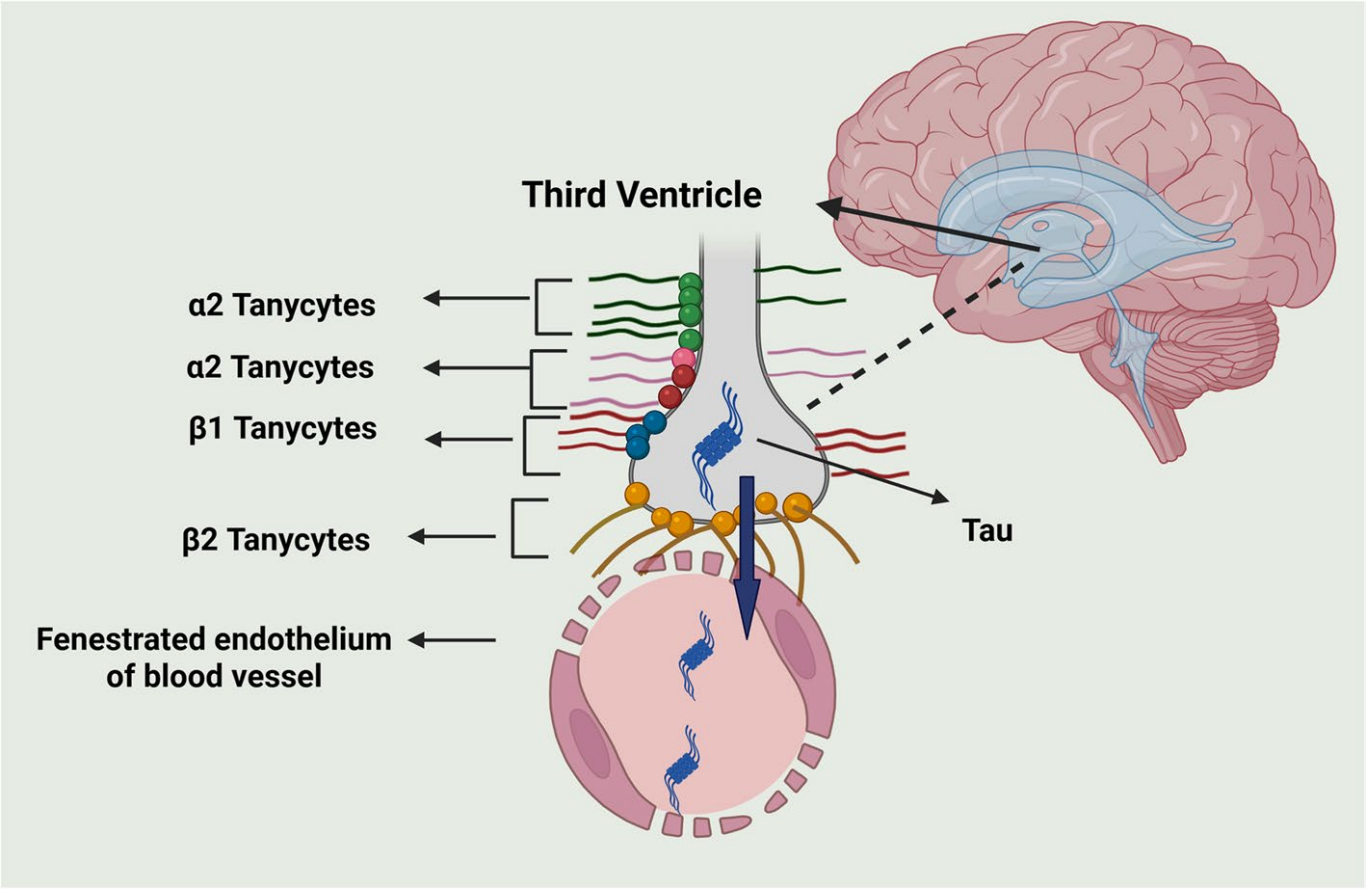
Role of tanycytes in tau clearance. Tanycytes translocate tau from cerebrospinal fluid in the ventricles towards the pituitary gland, where the portal capillaries of the median eminence were located. Created with BioRender.com

Tanycytes are glial radial cells lining the walls of the third ventricle and play crucial roles in neurogenesis, metabolic sensing, and maintaining brain homeostasis [122]. Their potential involvement in AD through mechanisms, such as compromised adult neurogenesis, abnormal metabolic signaling, and barrier control disruption of the median eminence, has recently become of interest. Tanycytes and CP are functionally connected by sharing roles in the homeostasis of brain fluid, molecular transport, and neuroendocrine regulation. The CSF secreted by CP carries signals affecting tanycytes, while tanycytes act as intermediaries for the delivery of hormones between CSF and the hypothalamus [123]. In addition, tanycytes exist in the blood-hypothalamic barrier, a part of the BBB consisting of vascular endothelial cells, pericytes, astrocytes, and basement membranes. The BCSFB could be involved with Aβ removal in aging by boosting Aβ outflow transporters and reducing inflow transporters. Tanycytes help protect the brain by moving harmful substances, like soluble Aβ and tau, from CSF into nearby blood vessels, allowing them to reach the pituitary gland and enter the bloodstream for removal (Fig. 5). Degradation of tanycytes in AD patients may play a role in the increased accumulation of tau in the brain by interfering with its transport [124]. However, in elderly AD patients, a compensatory mechanism, like BBB leakage, may account for the normal tau levels in CSF. Qi et al. recently summarized how alterations in tanycyte-originated neurogenesis following the modification of factors, such as BDNF, ciliary neurotrophic factor (CNTF), and insulin-like growth factors (IGFs), enhance neurodegeneration and cognitive decline in AD [23]. The involvement of tanycytic degradation in AD and the proof of a tanycytic transport system from the brain to the blood are remarkable, prompting inquiries about the role of tanycytes in clearing mechanisms and the onset of AD.

### Potential treatment strategies

Unlike classical AD, non-traditional AD (CP and BBB dysfunction) pointed to a different disease pathway, in which AD pathology may be impacted by impaired protein clearance rather than overproduction. Such patients may not respond well to the traditional AD treatments, like anti-amyloid and anti-tau medications. In such cases, a possible treatment option focusing on CSF clearance mechanisms (enhancing BBB or CP function) would be beneficial. Possible therapeutic interventions for CP dysfunction could be using anti-inflammatory therapies (CXCL-10 and TNFα inhibitors) and enhancing CSF clearance (aquaporin-4: AQP4 modulators; sleep-based therapies) to boost the glymphatic flow. AQP4 is a water channel protein in the CNS (mainly expressed on the end feet of astrocytes at the BBB)[125]. Since its main function is maintaining brain water homeostasis, AQP4 is an important component of the glymphatic system, aiding in the clearance of Aβ and tau protein from the brain. BBB dysfunction is categorized by loss of vascular integrity, protein leakage, and neuronal hypoplasticity. Therefore, restoring BBB integrity and endothelial functions (angiotensin receptor blocker, fibrin, and pericyte-targeting therapies) would ameliorate the vascular inflammation and protein leakage (TNFα and IL-1β inhibitors, lipid-lowering drugs, albumin replacement therapy), promoting neurovascular restoration (neurotrophic factor-based therapies, vascular endothelial growth factor: VEGF modulators) [126–129].

BBB or CP could be damaged by TNF-α through mechanisms like matrix metalloprotease (MMP)-mediated tight junction breakdown, increased barrier permeability, or inflammation-triggered structural changes [130]. These effects have been shown in cell models, including experiments involving TNF-α exposure (e.g., MMPs disrupting barrier integrity in CP epithelial cell lines) and similar effects at the BBB in vitro models with human endothelial cells [131]. Several evidence presented a direct role of cytokine signaling in CP dysfunction in AD and how CP function declined in aging and AD, affecting CSF production, toxic Aβ clearance, and immune gateway function to the CNS [132]. For instance, transcriptomic analyses of CP tissue from AD patients revealed upregulation of genes involved in acute-phase and inflammatory responses, cell adhesion, and chemotaxis, reflecting an altered immune environment at the blood-CSF interface [70]. During neuroinflammation, CP expresses specific adhesion molecules and chemokines that regulate leukocyte trafficking across the blood-CSF barrier, and elevated IFN-γ levels in CP have been shown to trigger macrophage recruitment into CSF [133]. In experimental APP/PS1 mice, TNF-α/TNFR1 signaling was elevated in the CP tissue and contributed to epithelial morphological damage and barrier disruption. Importantly, inhibition or deletion of TNFR1 restored CP integrity, reduced inflammation, and improved cognition in these models [134]. Together, these findings supported that CP-specific cytokine signaling, not just peripheral or parenchymal inflammation, played a mechanistically critical role in AD pathogenesis.

Murine model studies using anti-TNF-α monoclonal antibody, Infliximab, reported a reduction in Aβ plaques, tau phosphorylation [135], and cognitive deficits [136]. Other drugs, like Tocilizumab (IL-6 inhibitor) [137] and Anakinra (IL-1 inhibitor), were explored for their use in AD treatment. A recent study demonstrated the repurposed use of Anakinra in AD treatment, both in vitro (LPS-induced stress in BV2 cells) and in vivo (zebrafish model). Anakinra reduced ROS, nitric oxide production, along with downstream expression of *NF-κB*, *TNF-α*, *IL-1β*, *IL-6*, and *NOS* genes in BV2 cells. It also improved synaptic plasticity and neurogenesis in acrylamide-induced neurotoxicity in zebrafish larvae by increasing BDNF and CREB mRNA levels [138]. However, these therapeutics have not yet been systematically studied for barrier-specific AD models. Such models, which include tight junction markers, barrier electrical resistance, tracer permeability assays, or CP epithelial morphology, would be beneficial to differentiate between general anti-inflammatory effects from barrier-specific restoration.

Around 90% of the total circulating Aβ is predominantly bound to albumin [139], which creates a dynamic equilibrium between peripheral and central Aβ. Considering this fact, a new approach was developed to exchange plasma with albumin replacement (Alzheimer’s Management by Albumin Replacement: AMBAR program). For the AMBAR clinical trial, 41 hospitals across the USA (22) and Spain (19) took part. A total of 496 patients with mild to moderate AD were screened between the ages of 55 to 85 years [140]. Phase I focused on testing whether plasma exchange using 5% albumin could shift Aβ from the brain into the bloodstream and whether it had any effect on cognitive Function. Only 7 patients participated, each receiving 6 plasma exchange sessions over three weeks, followed by a year-long follow-up. The treatment successfully mobilized Aβ, and patients remained stable after a year [141]. In Phase II (N = 42), treated patients underwent 18 plasma exchanges over several weeks. This phase evaluated brain imaging and changes in cognitive, functional, and behavioral abilities. Results showed improvements in memory, language, and overall cognition, along with a drop in plasma Aβ levels in the treated group. In contrast, the control group continued to decline, as typically seen in AD [142]. Phase IIb/III expanded the study by testing different doses of albumin (5% and 20%) with or without intravenous immunoglobulin (IVIG). The group that received high-dose albumin combined with IVIG showed the most benefit, particularly in language and mental processing speed, suggesting a possible slowing or stabilization of disease symptoms [143, 144].

The CCL2/CCR2 axis is known to increase neuroinflammation, BBB disruption, and AD pathology. Targeting this pathway could provide novel opportunities for the treatment of AD [145, 146]. In a 2019 study, the effect of Reboxetine (a selective norepinephrine reuptake inhibitor) was evaluated in 5xFAD mice, which resulted in neuroprotective and anti-inflammatory changes after 28 days from the drug administration. It was also observed that deletion of CCL2 slowed down memory impairments, mitigated neuroinflammation, reduced Aβ plaque accumulation, and reduced degeneration of neurons in the brain cortex [147]. Similarly, the CXCL10/CXCR3 axis is also an active pathway in AD progression. A study reported that the repurposing of Treprostinil (vasodilator), Eplerenone (aldosterone antagonist), and Pitavastatin (HMG-CoA reductase inhibitor) significantly decreased CXCL10 secretions in N9 cells and primary microglia [148]. Enhancing Aβ clearance and BBB permeability using pericyte cells could prove crucial for future AD treatment [149]. Inhibitors, such as FER-1 (ferroptosis inhibitor), Mdivi-1 (mitophagy inhibitor) [150], Ghrelin [151], BQ-123 (endothelin receptor antagonist) [152], and anserine/carnosine [153] are a few pericyte-targeting or pericyte-associated signaling pathway-targeting inhibitors for AD treatment [154].

Stem cell therapy demonstrated that hPSC-derived pericytes can efficiently promote recovery and rescue of the BBB barrier function in the transient middle cerebral artery occlusion (tMCAO) mouse model [155]. While iPSC-derived brain pericytes can be used as a replacement therapy [156]. Customized AD therapy suggests the importance of subtype-specific potential biomarkers in identifying the mechanism of disease and its therapeutic intervention (Table 3).

**Table 3:** A summary of various possible treatments for CP/BBB dysfunction in AD

Strategy	Target Mechanism	Examples/Agents	Evidence	Ref
Anti-inflammatory therapy	Reduce CP/BBB inflammation	TNF-α inhibitors (Infliximab), IL-1 (Anakinra), IL-6 (Tocilizumab)	Infliximab reduced Aβ/tau pathology in murine model; Anakinra improved neurogenesis in vitro and zebrafish	[135–138]
Chemokine axis modulation	Block CCL2/CCR2 and CXCL10/CXCR3 pathways	Reboxetine, Treprostinil, Eplerenone, Pitavastatin	Reboxetine reduced inflammation, Aβ plaques; other agents decreased CXCL10 secretion in microglial cells	[145–148]
CSF clearance enhancement	Improve glymphatic flow, CP function	AQP4 modulators, Sleep-based therapies	AQP4 regulates Aβ/tau clearance via glymphatic system	[125]
Albumin replacement therapy	Enhance peripheral Aβ binding; promote clearance	5–20% Albumin ± IVIG (AMBAR trial)	Phase I–III trials: improved cognition, reduced Aβ in treated AD patients	[139–144]
Endothelial/vascular repair	Restore BBB integrity; reduce vascular leakage	Angiotensin receptor blockers, fibrin-targeting drugs, lipid-lowering agents	Improve vascular function, reduce neuroinflammation	[127, 128]
Neurovascular regeneration	Promote endothelial and neuronal health	VEGF modulators, BDNF-based therapy	Neurotrophic factors improve neurovascular integrity	[129, 138]
Pericyte-targeted therapy	Rebuild BBB via pericyte function	BQ-123, Mdivi-1, FER-1, Ghrelin, Anserine/Carnosine, stem cell–derived pericytes (hPSC/iPSC)	hPSC-derived pericytes restored BBB function in the stroke model	[149–153]
Amyloid/tau clearance enhancers	Enhance immune-mediated protein clearance	IL-34 (via CSF1R), Infliximab	IL-34 promotes Aβ clearance; Infliximab downregulates inflammatory markers	[37, 60, 126, 135, 136]
Stem cell therapy	Replace BBB/CP cells; repair neurovascular unit	hPSC/iPSC-derived pericytes	Recovered BBB integrity and function in in vivo models	[155, 156]

*Aβ* Amyloid beta, *AD* Alzheimer’s disease, *AMBAR* Alzheimer Management by Albumin Replacement, *AQP4* Aquaporin-4, *BBB* Blood–brain barrier, *BDNF* Brain-derived neurotrophic factor, *BQ-123* A peptidic endothelin ETA receptor antagonist, *CCL2* CC motif chemokine Ligand 2, *CCR2* CC chemokine receptor type 2, *CP* Choroid plexus, *CSF* Cerebrospinal fluid, *CSF-1R* Colony stimulating factor 1 receptor, *CXCL10* CXC motif chemokine Ligand 10, *CXCR3* Chemokine receptor of CXC, *FER-1* Ferroptosis inhibitor, *hPSC* Human pluripotent stem cells, *iPSC* Induced pluripotent stem cells, *IVIG* Immunoglobulin, *IL* Interleukin, *Mdivi – 1* Mitochondrial DIVIsion inhibitor 1, *VEGF* Vascular endothelial growth factor

## Conclusion

Understanding AD subtypes through their specific biomarkers would increase the precision of diagnoses, allowing for tailored interventions and aiding in the creation of novel therapies for subtypes that might not react favorably to conventional amyloid/tau-targeting strategies. Instead of using a “one-size-fits-all” strategy, more precise patient classification would result from the discovery of biomarkers specific to each subtype. Particularly for patients exhibiting an atypical course (clearance dysfunction) of AD, subtype-specific biomarkers may facilitate earlier and more precise diagnosis. Finding biomarkers implicated in endothelial integrity, CP function, or CSF clearance might be more beneficial as subtype-specific indicators in such cases. Upcoming clinical trials should evaluate these targeted methods that rely on biomarker-based patient categorization. The development of BBMs will allow a routine screening for AD pathology and will enable clinicians to understand the convenient and non-invasive markers of AD. The possible biomarkers discussed in this review have been primarily studied in the context of early diagnosis; their potential connection to CP/BBB dysfunction remains an emerging area that requires dedicated studies, particularly those incorporating imaging, proteomic profiling, or multi-omics approaches.

## Clinical trial number

Not applicable.

## Data Availability

No datasets were generated or analysed during the current study.

## Abbreviations

A2M: Alpha-2-macroglobulin
Aβ: Amyloid beta
ABCA7: ATP-binding cassette subfamily A member 7
AD: Alzheimer’s disease
AD7c-NTP: Alzheimer-associated neuronal thread protein
ADNI: AD Neuroimaging Initiative
AGE: Ansan Geriatric Study
AMBAR: Alzheimer’s Management by Albumin Replacement
Ang-1: Angiopoietin-1
APOC1: Apolipoprotein C1
APOE: Apolipoprotein E
APP: Amyloid precursor protein
AQP4: Aquaporin-4
BACE1: Beta-Secretase
BBB: Blood–brain Barrier
BBMs: Blood biomarkers
BCSFB: Blood-cerebrospinal barrier
BDNF: Brain-derived neurotrophic factor
BLSA: Baltimore Longitudinal Study of Aging
BV2 cell: Murine microglial cell line
CADS: Chongqing Ageing & Dementia Study
CCL: C–C motif chemokine ligand
CHI3L1: Chitinase-3 like 1
CLNK: Cytokine-dependent hematopoietic cell linker
CNTF: Ciliary neurotrophic factor
CP: Choroid Plexus
CPEC: CP epithelial cells
CREB: CAMP response element-binding protein
CSF: Cerebrospinal fluid
CSF1R: Colony-stimulating factor-1 receptor
CVO: Circumventricular organs
ECHDC3: Enoyl-CoA hydratase domain containing 3
ELISA: Enzyme-linked immunosorbent assay
FOXA2/PEN2/APP: Forkhead box protein 2/Presenilin enhancer protein 2/ Amyloid precursor protein
FWf: Free water fraction
GESTALT: Genetic and Epigenetic Signatures of Translational Aging Laboratory Testing
GFAP: Glial fibrillary acidic protein
GWAS: Genome-Wide Association Study
HC: Healthy control
HO-1: Heme oxygenase-1
hPSC: Human pluripotent stem cells
IGF: Insulin-like growth factors
IL: Interleukin
IMR: Immunomagnetic reduction
iPSC: Induced pluripotent stem cells
IVIG: Intravenous immunoglobulin
KARVIAH: Kerr Anglican Retirement Village Initiative in Ageing Health
KCL-ART: Alzheimer’s Research Trust cohort, Kings College London
LC–MS: Liquid Chromatography-Mass Spectrometry
LPS: Lipopolysaccharide
LRP-1: Lipoprotein receptor-related protein 1
MCI: Mild cognitive impairment
MCP-1: Monocyte chemoattractant protein-1
MMPs: Matrix metalloproteinases
MMSE: Mini-Mental State Examination
MR: Mendelian randomization
MRI: Magnetic resonance imaging
Nano LC: Nano-liquid chromatography-mass spectrometry
ND: Neurodegenerative disease
NfL: Neurofilament light chain
NFT: Neurofibrillary tangles
NF-κB: Nuclear factor kappa B
TNFα: Tumor necrosis factor alpha
NDEV: Neuronally derived extracellular vesicle
NO: Nitric oxide
NOS: Nitric oxide synthase
NTP: Neuronal thread protein
NVU: Neurovascular unit
PAI-1: Plasminogen activator inhibitor type 1
PDGFR-β: Platelet-derived growth factor receptor beta;
PET: Positron emission tomography
PICALM: Phosphatidylinositol binding clathrin assembly protein
PPMI: Parkinson’s Progression Markers Initiative
pTau: Phosphorylated Tau
PZP: Pregnancy zone protein
RAGE: Receptor for advanced glycation end products
RCAN1: Regulator of Calcineurin 1
REST: RE1-silencing transcription factor
RID: Radial immunodiffusion
RJNB-D: Ruijin NeuroBank of Alzheimer’s Disease and Dementia
ROS: Reactive oxygen species
RSS: Rotterdam Scan Study
SIMOA: Single molecular arrays
SUZ12: Polycomb repressive complex 2 subunit
TGF-β: Transforming growth factor beta
tMCAO: Transient middle cerebral artery occlusion
TMEM106B: Transmembrane protein 106B
tPA: Tissue plasminogen activator
tTau: Total Tau
TTR: Transthyretin
VEGF: Vascular endothelial growth factor
VSMCs: Vascular smooth muscle cells
WU-ADRC: Washington University Alzheimer’s Disease Research Center
ZO-1: Zonula occludens-1
